# Patients as research partners in preference studies: learnings from IMI-PREFER

**DOI:** 10.1186/s40900-023-00430-9

**Published:** 2023-04-07

**Authors:** Meredith Y. Smith, Rosanne Janssens, A. Cecilia Jimenez-Moreno, Irina Cleemput, Mireille Muller, Serena Oliveri, Gwenda Simons, Valentina Strammiello, Isabelle Huys, Marie Falahee

**Affiliations:** 1 Evidera, Inc, PPD, a Part of Thermo Fisher Scientific, 6 Plainfield Street, Boston, MA 02130 USA; 2 Department of Regulatory and Quality Sciences, School of Pharmacy, University of Southern California, Los Angeles, CA USA; 3 Department of Pharmaceutical and Pharmacological Sciences, KU Leuven, Leuven, Belgium; 4 Evidera-PPD, London, UK; 5 Translational and Clinical Research Institute, Newcastle, UK; 6 Belgian Health Care Knowledge Centre (KCE), Brussels, Belgium; 7 Regulatory Affairs, Novartis, Basel, Switzerland; 8 Applied Research Division for Cognitive and Psychological Science, IEO, European Institute of Oncology IRCCS, Milan, Italy; 9 Rheumatology Research Group, Institute of Inflammation and Ageing, University of Birmingham, Birmingham, UK; 10 European Patients’ Forum, Brussels, Belgium

**Keywords:** Patient preferences, Patient research partners, Patient preference study, Patient involvement, Patient and public involvement/engagement (PPI), Patient impact, Medical product decision-making

## Abstract

**Background:**

There is growing recognition of the importance of patient and public stakeholder involvement (PPI) in patient preference research. However, limited evidence exists regarding the impact, barriers and enablers of PPI in preference studies. The Innovative Medicines Initiative (IMI)-PREFER project conducted a series of preference case studies which incorporated PPI.

**Objective:**

To describe: (1) how PPI was operationalized in the PREFER case studies, (2) the impact of PPI, and (3) factors that served to impede and facilitate PPI.

**Methods:**

We reviewed the PREFER final study reports to determine how patient partners were involved. We conducted a thematic framework analysis to characterize the impact of PPI and then administered a questionnaire to the PREFER study leads to identify barriers and facilitators to effective PPI.

**Results:**

Eight PREFER case studies involved patients as research partners. Patient partners were involved in activities spanning all phases of the patient preference research process, including in study design, conduct and dissemination. However, the type and degree of patient partner involvement varied considerably. Positive impacts of PPI included improvements in the: (1) quality of the research and research process; (2) patient partner empowerment; (3) study transparency and dissemination of results; (4) research ethics, and (5) trust and respect between the research team and the patient community. Of the 13 barriers identified, the 3 most frequently reported were inadequate resources, insufficient time to fully involve patient partners, and uncertainty regarding how to operationalize the role of ‘patient partner. Among the 12 facilitators identified, the two most frequently cited were (1) having a clearly stated purpose for involving patients as research partners; and (2) having multiple patient partners involved in the study.

**Conclusion:**

PPI had many positive impacts on the PREFER studies. Preference study leads with prior PPI experience reported a greater number of positive impacts than those with no such experience. In light of the numerous barriers identified, multi-faceted implementation strategies should be considered to support adoption, integration and sustainment of PPI within preference research. Additional case studies of patient partner involvement in preference research are needed as well to inform best practices in this area.

**Supplementary Information:**

The online version contains supplementary material available at 10.1186/s40900-023-00430-9.

## Abstract (summary)

Research about patients’ preferences for medicinal products and treatments is growing. Such research could be improved if patients were involved as ‘research partners,’ that is, as active members of the study team itself. To date, however, little is known about the actual experience of involving patients as partners in such research. This paper presents learnings from involving patients as partners in 8 case studies conducted as part of IMI-PREFER, a big, European-based project which aimed to develop recommendations about how to conduct preference research. Involving patients as partners led to improvements in the: (1) quality of the research and research process; (2) recruitment of participants; (3) content and design of patient-facing informational materials; and, (4) how and what study results were shared with patient communities. Our findings showed that it is important to plan for patient partners’ involvement early on in the design of the preference study so as to ensure that they are fully integrated into the research team and their opportunity to contribute to all stages of the research is optimized. Such planning should address how patient partners will be paid, what their role responsibilities will include, how and when they will be trained and educated, and how they will be supported throughout the course of the study. Having a clearly stated purpose for involving patients as research partners, selecting patient partners who have had prior research experience and relationships with the researchers, and having multiple patient partners on the study team are all also helpful in supporting successful patient involvement. We need more people to share their experiences with involving patient partners in preference research so that we can continue to improve how this is done.

**Supplementary Information:**

The online version contains supplementary material available at 10.1186/s40900-023-00430-9.

## Introduction

Integrating patient values and perspectives into the benefit-risk assessment of medicines is a key goal for the advancement of drug development in the twenty-first century [1–4]. Consistent with this goal, there has been a marked proliferation over the past decade in the number of studies assessing patients’ preferences and perceived trade-offs regarding medicinal treatments and their attributes [5–11].

One accelerant in this regard was the Innovative Medicines Initiative—Patient Preferences in Benefit-Risk Assessments during the Drug Life Cycle (IMI-PREFER) project [12]. PREFER was a multi-stakeholder partnership among academia, Health Technology Assessment (HTA) bodies, industry, patients and patient organizations, and regulators. It had two central goals: (1) to determine how and when patient preferences are of value for decision-making during the medicinal product life-cycle, and (2) to formulate recommendations on patient preference research for informing industry, regulatory authorities and health technology assessment (HTA) decision-making. To address these goals, PREFER sponsored a series of case studies in different disease areas. The PREFER core case studies were not designed to directly inform specific decisions, but rather to address certain clinical and methodological research objectives identified by PREFER. At its conclusion in May 2022, PREFER had produced a formal set of recommendations, a positive qualification opinion from the European Medicines Agency and numerous scientific articles and webinars [12].

Commensurate with advancements in the science of patient preference research, there has been growing awareness of the importance of partnering with patients in the conduct of health-related research, both generally and in the context of patient preference research specifically [13–16]. Partnering with patients entails doing research “with” as opposed to “for” them and the concept has been popularized under the rubric “patient and public involvement” or PPI [17]. Involving patients, as well as other public stakeholders, in healthcare research has been recognized as vital for many reasons, including ensuring that the research is relevant and that findings can be translated into policy and/or practice [13, 18]. This recognition has spurred demand for research that involves patients as partners and has led to a growing body of empirical research describing when and how to do so [14–16, 19–30].

Consistent with this trend, PREFER also emphasized the inclusion of patients as research partners. In PREFER, PPI was defined as “a patient, informal caregiver or patient advocacy organization representative with experience or knowledge of the disease, who served as a member of the research team.” PPI was an expectation for the three PREFER core case studies; however, decisions regarding how and when to involve patient partners, and to what extent, were left to each core case study team to determine. In contrast, PPI was encouraged, but not mandated, for the other, non-core case studies.

Despite the growing interest in involving patients as preference research partners [14], and recognition of the many ways in which they can contribute to the research endeavour [22, 23], there are limited examples of experience-based insights regarding the impact of involving patients in this way [1, 22]. Indeed, Shields and colleagues [22], in a recent review of the published preference literature, concluded that **“**Despite growing recognition of the potential benefits of patient and public involvement (PPI) and the formal requirement by many funders to include PPI in research, we found *a limited number of preference studies that utilized PPI activities***”** (italics added) [22].

In this paper, we sought to address this gap in knowledge by extracting learnings from patient preference case studies conducted as part of the PREFER project. Specifically, we examined researcher reports on the following questions: (1) How were patient research partners involved in the PREFER case studies, (2) What was the impact of PPI, and (3) What were the factors that served as barriers and facilitators to PPI? Data were gathered through analysis of the final reports of PREFER case studies as well as via follow-up questionnaires with PREFER case study leads. Both data sources were derived from the perspective of the lead scientific study investigator (the “study lead”) of each case study.

## Materials and methods

### Data sources

Study data were drawn from two sources. The first source was the final study reports from the eight case studies conducted under the aegis of the IMI-PREFER initiative which had involved patients as research partners. Of these, 3 were led by research teams funded by PREFER, and were referred to as the “core” case studies. They focused on the following disease areas: (1) non-small cell lung cancer (NSCLC); (2) preventive treatment of rheumatoid arthritis (RA Preventive Treatment); and (3) neuromuscular disorders (NMD). The remaining five case studies were funded through sources external to PREFER. They included three academic-led case studies, one focussing on RA, another on gene therapy for haemophilia, and a third focussing on multiple myeloma (MM). The remaining two studies were industry-led and included one focussing ondiabetes and a second on chronic obstructive pulmonary disease (COPD).

The primary goal of these reports was to serve as the definitive documentation of each case study in terms of purpose, design, methods, and results. As part of that documentation, the final reports were also required to include a paragraph describing how and to what extent patients were involved as research partners. They were prepared by the case study principal investigators (case study “leads”) with assistance from other study team members and followed a format pre-specified by the PREFER consortium. Each report was required to include a dedicated paragraph describing the activities and impacts associated with PPI.

A second source of study data came from a follow-up questionnaire on barriers and facilitators to PPI. In addition to describing different forms of PPI and associated impacts, several of the case study reports also mentioned factors that had hindered or enabled PPI. We conducted a thematic analysis of these factors to generate a checklist. We supplemented this list of potential barriers and enablers with factors identified from the literature on patient involvement in health research [14, 21]. We then incorporated the final checklist into a follow-up questionnaire. This questionnaire was sent to the 8 case study leads with a request for them to complete it. Questionnaire items included: the number of patient research partners involved in the case study, a list of types of activities performed by them, the ‘added value’ of PPI, a checklist of potential barriers and facilitators to PPI (with a free-text box for nominating additional factors), and 3 open-ended questions regarding the added value of PPI, what they might have done differently to facilitate patient involvement, and how, if at all, patient research partners were compensated. [see Additional file 1 for copy of Follow-up Questionnaire].

### Review and coding of data on impacts, barriers and enablers to patient involvement

We conducted a thematic assessment of impacts, barriers and enablers based on information extracted from the final case study reports. We used the Framework Method to guide our assessment as it is a systematic and pragmatic approach to the identification of key patterns across cases and has been widely applied to analyze qualitative data [31–33]. The Framework Method consists of a multi-step process involving data review, coding and data charting into an analytic framework matrix for analysis and interpretation. The individual case study was used here as the unit of analysis. Codes were then developed manually by three experienced qualitative researchers based on an initial, independent reviews of the three core cases (MF, GS, MYS) in Microsoft Excel^®^ (Microsoft Corp., Redmond, WA, USA). Assigned codes were then compared and discussed to reach a consensus on a provisional code list. The accuracy and consistency of the coding across data were assessed by another researcher (RJ). Concepts were captured in a data extraction table.

### Data analysis

We conducted a descriptive, ad hoc analysis of each case study final report to determine how patients were involved as research partners and to characterize the study leads’ perspectives regarding the impact of PPI. We used two different frameworks to guide our analysis of study data. First, we used the PREFER patient involvement in research framework as the basis for systematically characterizing the types of PPI activities performed in each case study (see Additional file 2: Fig. S1) [16]. We selected this framework because it had been specifically designed to describe the stages of and activities involved in conducting preference research that present opportunities for PPI and was developed in collaboration with patient research partners and other stakeholders as part of the PREFER initiative [12]. The framework consists of 3 components: Component 1 refers to the development of the study purpose and objectives; Component 2 refers to the organization, design and conduct of the preference study; and Component 3 to the dissemination and application of study results to inform industry, regulatory and HTA decision-making.

Second, we used the PCORI patient involvement impact framework to map each of the (see Table 1) [19]. The PCORI impact framework was selected due to its applicability for the purpose of mapping impacts and its rigorous development process (see prior section on data review and coding for a description of the thematic analysis that was used to identify the range of impacts).

**Table 1:** Map of potential versus reported impacts of patient research engagement by PREFER case study^a^

Hypothesized dimensions of impact to measure			Actual	Impacts	Reported			
	COPD	Gene therapy	Hemophilia	MM	NMD^b^	NSCLC^b^	RA	RA preventive treatment^b^
*I. Better quality research*								
1. Research quality and research process: enhanced credibility and improved research	X	X	X	X	X	–	X	X
2. New funding and funding opportunities: patient contributed to research proposal	–	–	–	–	–	–	–	X
3. Research questions (e.g., which treatment attributes to assess), hypotheses, interventions and medical technologies become more relevant/usable for patients	X	X	X	X	X	–	X	X
4. Research design, methods and study procedures become more appropriate, sensitive and/or ethically acceptable	X	X	X	X	X	–	X	X
5. Recruitment, accrual rates, and retention improvements	X	–	X	X	X	X	–	X
6. Representativeness/diversity of research subjects (i.e., inclusion of more hard-to-reach patients)	–	–	X	–	X	–	–	–
7. Data collection procedures and data quality changes	X	X	X	X	X	–	X	X
8. Intervention and/or survey implementation by patients	–	X (piloting)	–	–	X (piloting)	–	X (piloting)	X (pretesting)
9. Data analysis and/or results interpretation by patients	–	–	X	X	–	X	X	X
10. Researchers' knowledge and capacity increases	–	–	–	–	X	–	–	–
11. Changes in researchers' attitudes about the value of the patient perspective	–	–	–	–	X	–	–	–
12. More useful evidence for clinical and health policy decisionmaking	–	–	–	–	X	–	–	–
13. More relevant evidence for entire spectrum of patients with targeted disease condition	–	–	–	–	X	–	–	–
14. Changes to health outcomes, including overall population health, morbidity and mortality	–	–	–	–	–	–	–	–
***II. Other impacts***	–	–	–	–	–	–	–	–
1. Patient empowerment (a) Patient or community research knowledge, skills and capacity (b) Knowledge of community needs (empathy), services available, motivation to help community (citizenship literacy)	–	–	–	–	X (a and b)	–	–	–
2. Increased translation, dissemination and uptake of results via improved dissemination to patients and the community	–	X	–	X	X	X	–	X
3. Democracy and accountability (a) Transparency (b) Legitimacy (c) Accountability (d) Public trust in public institutions	–	(a) X (lay language summary)	–	(a) X (lay language summary)	(a) X (lay language summary)	(a) X (lay language summary)	–	(a) X (lay language summary)
4. Moral obligation (a) Fairness (b) Respect and trust between researchers and engaged stakeholders (c) More ethically acceptable research	–	–	–	–	(a, b, c) X	–	–	–
Total # of types of impacts reported	5	7	7	8	15	4	6	10

^a^Potential impacts adapted from PCORI framework by Esmail et al. [19]

^b^ = PREFER core case study

X = Impact reported

– = No impact reported

## Results

### How were patients involved as research partners in the PREFER case studies?

As presented in the table in Additional file 3, the number of patient partners varied by case study, ranging from 2 to 9. There was considerable variability as well in the degree to which the leads for the 8 case studies had prior experience in involving patients as research partners, ranging from no such experience (e.g., NSCLC case study) to extensive prior experience (RA Preventive Treatment case study).

The types of activities patient partners performed spanned all stages of preference research. The majority of activities, however, were concentrated in tasks related to study execution (e.g., development of the informed consent document, preference question design, sample definition and recruitment, data collection and analysis, report preparation, and dissemination of results), fully mapping onto Component 2 of the Patient Engagement Framework (see Additional file 2: Fig. S1). In only two instances (RA Preventive Treatment and MM case studies) were patient partners reported to have been involved in the determination of the study’s purpose (Component 1 in Additional file 2: Fig. S1), and there were no reported instances of patient partner involvement in applying results for the purposes of decision-making (Component 3 in Additional file 2: Fig. S1). Patient partners participated in research team meetings and teleconferences (in-person prior to the onset of the COVID-19 pandemic, and virtually thereafter), the PREFER annual meetings and in a patient involvement workshop in May, 2021. Throughout the course of each case study, patient partners co-developed and/or reviewed study documents and other study materials, and provided their input both in-person and via email.

### What was the impact of PPI?

There were multiple impacts associated with PPI across all 8 of the case studies. The number and type of impacts varied from study to study (Table 1). Impacts most frequently identified were those related to the quality of the research itself, including improvements in the relevance of the research questions posed, and in the appropriateness and sensitivity of the study methods and procedures. For example, in the Diabetes case study, patient partners helped generate ideas for treatment attributes to be included in the preference study, and designed and tested the patient educational materials describing those attributes. In the NMD case study, patient partners helped improve study quality by pre-testing the patient preference questionnaire to ensure that item wording was clear and understandable to patients, and that the formatting was correct. In the RA Preventive Treatment case study, patient partners revised the description of both the disease and the hypothetical treatments presented to study participants, and reviewed and revised the informed consent form, participant information sheets and background questionnaire to improve their readability, They also helped develop the survey questionnaire, including contributing to the construction and wording of the choice task scenarios and the selection and presentation of study attributes.

In the haemophilia study, where the level of disease and treatment expertise among patient partners was high, their feedback regarding the content of an educational video for study participants (which was quite technical and advanced in its’ coverage of the subject matter) improved its accessibility to haemophilia patients. In the RA Preventive Treatment study, patient partners’ input led to the removal of a diagram of joint erosion from the background information given to patients as it was deemed to be too ‘medicalized’ and ‘depersonalized’ and could potentially lead to participant disengagement with the material. Patient partners also added examples to the background information of how RA symptoms affected daily activities. Not least, patient partners assisted in dissemination of study results. For example, in the MM study, patient partners were instrumental in disseminating study results back to the patient community. They also co-authored the seminal study manuscript and assisted in developing abstracts and posters for professional conferences.

While no undesired forms of impact were identified, there were several additional positive types of impact that were seldom reported. For example, patient partner involvement in funding opportunities/applications was only reported by one study (RA Preventive Treatment). Other types of rarely reported impacts included: (1) improving researcher knowledge and attitudes regarding involving patients as research partners, (2) improving the usefulness of the study evidence for a diverse range of patients (i.e., with NMD and MM), (3) helping patient partners to feel more empowered, and (4) enhancing the sense of respect, trust and fairness between the scientific team members and the patient research partners. The study lead for the NMD study observed that working with patient partners had deepened her appreciation as a researcher regarding the value of partnering with patients: *“…. in the NMD study, I saw first-hand how valuable patient partners are and the unique contributions they make to improving the study design and assisting with interpretation of the study results.” (*NMD Case study report).

### What factors served as barriers or facilitators to PPI?

#### Barriers to PPI

Thirteen different barriers to PPI in the PREFER case studies were corroborated in the questionnaire responses (Table 2). There was substantial inter-study variability in the type and number of barriers mentioned. The most frequently mentioned barrier was inadequate resources to support PPI (n = 7; e.g., lack of dedicated budget to pay for patient partners’ reimbursement; no provision of formal on-boarding, training, and/or other sources of support).

**Table 2:** Barriers for involving patients as research partners by PREFER case study

	COPD	Gene therapy	Hemophilia	MM	NMD^a^	NSCLC^a^	RA	RA preventive treatment^a^
Barriers*								
CONFIDENCE					X			
CONTENT	X				X			
GEOGRAPHIC	X							
IMPRECISE						X		
PANDEMIC		X		X		X		X
PLAIN LANGUAGE					X			
RECRUITPAG	X		X					
SUPPORT		X	X	X	X	X	X	X
TIME			X			X	X	
UNCERTAIN				X	X	X		
OTHER	(1) In one of the participating countries, the patient advocacy group had no previous experience of this kind and hence their input to the project was limited (2) The language barrier was also a complicating factor in one country (Japan) requiring an intermediary in the interaction	"…We had a big group of patient research partners but perhaps not fully representative (e.g. only 1 male and no PRP from one of the participating countries)"						

Definition of Barrier Codes

CONFIDENCE: Patient partners felt intimidated or pushed out sometimes (either by discussion topics or other team members)

CONTENT: Patient Research Partners (PRPs) lacked sufficient content knowledge to contribute fully

GEOGRAPHIC: Geographical limitations for patients to participate as Patient Research Partners (PRPs)

IMPRECISE: The role of the PRP was not clearly defined/no job description provided and no rules of engagement were presented

PANDEMIC: COVID-19 pandemic interfered with study execution and/or PRPs ability to contribute to study

PLAIN LANGUAGE: Team used technical/medical research terminology as opposed to a common 'plain language'

RECRUITPAG: Having to rely on the Patient Advocacy (PAG) for recruitment purposes—i.e., this caused delays in recruitment; PAGs were not always responsive; in some instances, they unable to recruit a sufficient number of patients

SUPPORT: Inadequate resources were allocated (e.g., funding to pay for PRPs; formal on-boarding; training; other sources of support)

TIME: Insufficient time to get Patients as Research Partners fully involved- focus was on getting the research project up and running

UNCERTAIN: There was uncertainty [i.e., within the study team] regarding how to practically operationalize the role of patient as Research Partner (PRP)

OTHER: Any other factor that served to hinder the study team from involving patients as research partners

^a^PREFER core case

Other frequently mentioned barriers included lack of sufficient time to involve patients as research partners (n = 3), the COVID-19 pandemic (n = 4), and uncertainty regarding how to operationalize the role of the patient as research partner, including lack of a formal description of the patient partner role (n = 3). Reliance on patient advocacy groups (PAGs) for help with recruiting study participants was mentioned in both the COPD and haemophiliac studies as a barrier due to the fact that PAGs were not always able to assist fully in meeting the recruitment targets.

Other less frequently mentioned barriers included the use of patient-related study materials that were not written in plain language (n = 1), lack of sufficient disease and research-related knowledge on the part of some patient partners (n = 1), structuring of patient involvement opportunities in a way that was not supportive to patient partners (n = 1), geographic limitations for patient partners (e.g., to travel to study-related meetings, n = 1), language barriers between patient partners and other team members (n = 1), lack of full representation of the patient population (n = 1), and insufficient research experience on the part of some PAGs involved in the studies (n = 1). Lack of confidence was also mentioned as a barrier in one case study (NMD). The NMD case study lead observed that both a patient and caregiver research partner expressed a lack of confidence in participating based on a perceived power differential between them and the other research team members. As a result, we renamed the “power” barrier (as specified in the questionnaire) into a “confidence” barrier to more accurately reflect the specific factor at play. No data were reported on 3 of the questionnaire barriers: Resist (i.e., researchers resisted or undermined patient partner involvement), Dynamics (i.e., research team group dynamics were negative/dysfunctional), and Tension (i.e., there was tension between the scientific research leads and the patient partners).

#### Facilitators to PPI

Twelve facilitating factors to PPI were mentioned (Table 3). As with barriers, there was substantial inter-study variability in the type and number of facilitating factors identified. The two most frequently cited facilitating factors were (1) having a clearly stated purpose for involving patients as research partners; and (2) having multiple patient partners involved in the study.

**Table 3:** Facilitating factors for involving patients as research partners by PREFER case study

	COPD	Gene therapy	Hemophilia	Multiple myeloma	NMD^a^	NSCLC^a^	RA	RA preventive treatment^a^
Facilitators^a^								
CONTRIBUTE	X			X	X			
EXPERIENCE	X	X	X	X	X			X
MECHANISM	X	X	X	X	X		X	
MEETING	X			X		X	X	
OWNER	X			X				
PAY	X		X		X		X	
PURPOSE	X	X	X	X	X	X	X	X
RELATION	X		X	X	X	X		X
REPRESENT	X	X	X	X	X		X	X
RECRUIT	X		X	X	X	X	X	
TIME	X			X				X
SUPPORT	X			X	X		X	
URGENT		X		X			X	

Definition of Enabling Factors' Codes

CONTRIBUTE: Patient contributions as PRPs were encouraged/recognized by the research team

EXPERIENCE: PRP had participated in prior research studies

MECHANISM: The research project work was organized in such a way as to ensure that the patient's voice was incorporated

MEETING: Research team meetings were scheduled to accommodate PRPs

OWNER: There was a clear sense of 'co-ownership' between PRPs and research team members in terms of the research agenda

PAY: Patients as Research Partners (PRPs) received some type of financial compensation for their work. PURPOSE: Clearly stated purpose for involving Patients as Research Partners

RELATION: Presence of existing (informal) relationships were in place between research team members and the patients who served a Patient Research Partners (PRP)

REPRESENT: There was more than 1 Patient as Research Partner (PRP) involved in the study

RECRUIT: Patients as Research Partners (PRPs) were recruited via networking with existing patient stakeholder groups

SUPPORT: Principal investigator of study ensured that PRPs received information, training to support them in their role

TIME: There was sufficient lead time to involve/recruit patients as PRPs

URGENT: There was a sense of urgency about the importance of doing the research (i.e., with patient involvement as research partners)

^a^PREFER core case study

One cluster of facilitators concerned the experiential qualifications of the patient partners(s). PPI was facilitated when the patient partner had had prior experience working in the research partner capacity (n = 5), had a pre-established relationship with one or more of the scientific team members (n = 5), and was recruited as the result of networking with PAGs (n = 5).

Other important facilitating factors were related to how the patient partner was prepared, supported and recognized in his/her role as a patient partner. Facilitating factors of this type included the provision of support (e.g., training, resource planning guidance, information) (n = 4), meetings (n = 4), the structuring of the research project work so as to ensure that the patient partner’s voice was incorporated (n = 4), encouragement and recognition of patient partner contributions by others on the research team (n = 3), having sufficient time to engage and train the patient partner (n = 3), having a sense of urgency about the importance of doing the research with patient partner involvement (n = 3), having a clear sense of ‘co-ownership’ of the research agenda between the patient partner and others on the study team (n = 2), the provision of compensation to the patient partner (n = 3), and upfront specification of the patient partner role and responsibilities (n = 1).

#### Reflections regarding what could have been improved to support PPI in patient preference studies in the future

The questionnaire also asked study leads to reflect on what they might have done differently to improve the practice of PPI in the PREFER case studies. Several themes emerged, including a constellation of issues related to how the role of patient partner was defined and practically operationalized within each study. One case study lead emphasized the importance of clearly defining the role of the patient as research partner—ideally before study onset. “*I would practically operationalize the role of patient as research partner*” (NSCLC). Related to this was issue of how to operationalize the role of patient partner in practical terms an issue which correlated with the “Uncertain” barrier identified in the barriers assessment. A study lead noted that “*with respect to the uncertainty regarding practical organization, we learned along the way how to practically involve them* [patient partners] *(regular meetings, asking for structured input). I think all these are important points that deserve focus in future PPS [patient preference study]-patient involvement activities*” (MM case study lead).

Other reflections concerned the importance of communicating expectations upfront, providing more opportunities for PPI and offering better support. The NMD study lead noted that, in hindsight, she would have established “*clearer communication lines since the beginning; Know in advance that they would be invited to investigators meetings so they could also prepare in advance; Have given them the chance to speak at a panel or something at the investigators meeting; Better and standardised educational materials at the beginning.”*

The NSCLC study lead stated that she would have allocated more resources to involving patients*,* while the NMD study lead noted that she would have ensured that a payment structure for patient partners was in place from the beginning.

A recurrent observation concerned the importance of involving patient partners early on at the very start of the project: “*I would have involved patient research partners earlier in the project. In writing the aim and selecting the research methods.*” (RA Preventive Treatment case study lead).

Another observation related to the importance of having sufficient numbers of patient partners involved so that if some opted to discontinue, the work could continue uninterrupted. Failure to anticipate patient partner turnover, as the NMD study lead noted, “…*complicate[d] matters as we had then to retrain new persons who had not been part of the initial discussions.*”

Reliance on PAGs as sources for recruiting patient partners was also cited. This reliance was noted as barrier as well in that exclusive reliance on PAGs for recruitment purposes often resulted in delays or outright shortfalls in reaching study recruitment quotas. An upfront appraisal of the PAG’s capabilities to do so and of the research team’s ability to support patient partners fully was cited as being important to ensuring a satisfactory partnership. As the study lead of the COPD study observed, “*the choice of the patient support group …. was not ideal, we should have looked more widely before selecting the patient support group there*.”

An additional theme concerned the tension between completing the case study on time and the time needed to engage with the study patient partners, especially when the latter’s availability was limited. This, too, was noted as a barrier. As the Gene Therapy study lead noted, “*We would have loved to involve the patient partners in recurring meetings of the research team, but because of limited time that they had available we had separate meetings with them. In the future, if possible, I believe it would be beneficial to include them more regularly in research team meetings.*” Similarly, the MM study lead wished that the team had had “*more time available to involve them in an iterative way (maybe including even more iterations during the survey development), so that the study timelines can accommodate their availability for input.*”

Lastly, there was an observation about emphasizing the importance of including patients as research partners. The MM study lead wished that she had been more vocal about “*…. making clear towards other study partners, patients and patients organisations the value that patients and patients organisations bring.*”

## Discussion

Our study adds to the small but growing literature on the impact of PPI in the context of patient preference research specifically, as well as to the larger literature on PPI in health research more generally. Results show how PPI can be implemented in patient preference research in a range of different diseases and among different patient and caregiver populations. In addition, they offer practical insights regarding how to structure and operationalize PPI in order to optimize its impact, identify key barriers to anticipate, and provide suggestions on how several of these barriers might be addressed. Results also highlight an aspect of PPI that is unique to patient preference research—that is, the role that patient partners can play in identifying appropriate treatment attributes to include in the preference choice tasks, and in co-developing educational materials for study participants regarding these attributes.

The implications of our findings for PPI in patient preference research are three-fold. First, they show that patient partner involvement is feasible across all phases of the patient preference research process. Second, findings demonstrate that such involvement can yield a range of positive impacts. These impacts include improvements in: the quality of the research (e.g., defining the research question; developing the study design, recruiting patients, and preparing patient-facing study materials), patient partner empowerment (e.g., via acquisition of research knowledge, skills and experience); study transparency and dissemination of results; research ethics (e.g., via enhancing the ethical acceptability of the research); and the connection between the researchers and the relevant patient community (e.g., via fostering a greater sense of respect and trust between both parties). Third, they outline some best practices to follow when including patient partners in preference research, including clearly defining the purpose of and expectations for the role of patient partner up front, providing training and ongoing support to patient partners, having them participate in regular team meetings, and establishing strong lines of communication.

We contextualized our findings on impact with information on factors that served to impede and facilitate patient partner involvement in research. Among the most frequently cited barriers was that of uncertainty regarding how to operationalize the role of ‘patient partner.’ One manifestation of this uncertainty can be seen in the variety of ways that PPI was operationalized across the case studies, not only in terms of the number and types of activities patient partners were invited to engage in but how they were integrated into the larger research team and their role in project decision-making. For example, in the Gene Therapy study, patient partners contributed in relatively circumscribed ways (e.g., reviewing wording for patient-facing materials; pilot testing the patient survey; co-writing the lay language summary) and attended separate meetings from those held for the rest of the research team. In the MM study, expectations for the patient partner role evolved over time as the research team gained experience working with them, leading eventually to the establishment of regular meetings and the provision of structured guidance on how patient partners were to provide their input. Finally, in the RA Preventive Treatment study, patient partners were involved at project inception, engaged in a diverse array of activities and invited from the onset to attend the regular research team meetings.

Operationalizing the patient partner role entails more than defining its associated responsibilities (e.g., specific activities to engage in, types of deliverables to provide, timelines, etc.) and rights (e.g., rights to be reimbursed for expenses incurred due to participation) [34]. Arnstein posits an 8-tier typology or “ladder” of citizen participation ranging from ‘rubber stamping’ at the bottom to full accountability for project leadership at the top [34]. A participant’s position on the ladder is determined not only by the type of activities s/he performs, but the degree to which his/her input is heeded*-* i.e., is acknowledged and acted upon. Neither one of our study data sources provided comprehensive information on the level of decision-making and accountability patient partners exercised. Future PPI research should seek to collect such data in order to characterize the patient partner role more fully.

Another commonly reported barrier for patient partner involvement, that of inadequate funding, affected PPI impact as well. Insufficient financial support for patient partners is hardly unique to the PREFER studies; rather, it has been noted in several reviews of PPI [21, 22]. Budget limits can curtail the amount of staff time available to train and support patient partners, two factors that can substantially determine how effectively they perform their role and thus, the quality and quantity of PPI impact. In the PREFER core case studies, funds were allocated upfront specifically for PPI and a policy and procedures were established to support timely payments to patient partners. This represents a promising model to adopt in future preference studies featuring PPI.

Collectively, these findings emphasize the importance of comprehensively planning for patient partners’ involvement early on in the study conceptualization phase so as to ensure that they are fully integrated into the research team and their opportunity to contribute to the research is optimized. Such planning should include developing a process for compensating patient partners, budgeting for their reimbursement, preparing a formal role description, allocating time in the project schedule to train and educate patient partners, and providing ongoing support to them throughout the study’s duration.

Key among the facilitating factor was that of having a clearly articulated purpose for involving patients as research partners, a finding that is consistent with other PPI research [16, 21]. Clarifying the purpose of patient research partner involvement is related to operationalizing the patient role in that it serves to given him/her a recognized position within the team and a defined set of contributions, both of which can promote effectiveness, confidence and a feeling of being valued [35]. Moreover, clarifying the purpose of patient partner involvement can breed a greater sense of trust between both parties and increased commitment to the research project at hand [35].

Another facilitating factor included selecting patient partners who had prior research experience and who had pre-existing relationships with the researchers. A concern expressed in the PPI literature is that of ‘professionalization’ of the patient partner as a result of training, research experience and research team socialization [36, 37]. The central thesis of the patient professionalization argument is that, by virtue of having more training and experience, a patient compromises his/her ability to represent the patient perspective authentically [36]. In this instance, however patient professionalization may not be the issue so much as the fact that the patient partner represented a ‘known entity,’ one who had an established track record of working collaboratively with the researcher and who, by virtue of his/her previous training and experience, would therefore require less support and guidance in successfully fulfilling the patient partner role. The highest number of impacts was reported in the NMD and RA Preventive Treatment studies where the case study leads not only had pre-existing relationships with patient partners but were themselves highly experienced in patient partnering, were familiar with following good practice guidelines on patient research partner involvement, and had articulated a clearly stated purpose for patient partner involvement [38–41].

Having multiple patient partners on the study team was yet another facilitating factor which was mentioned. Each of the PREFER case studies had a minimum of 2 patient partners; some (e.g., MM and RA Preventive Treatment case studies) had many more. Our results suggest that the number of patient partners involved may also affect the type and degree of PPI-associated impact. It is not evident, however, what the optimal number of patient partners should be per study in order to maximize PPI impact. Clearly, there are trade-offs to consider: as the number of patient research partners increases, study impacts would be expected to increase; however, commensurate with this, both study timelines and budget costs (i.e., to cover increased patient partner reimbursement and additional staff hours for training and support) would be expected to rise as well.

## Limitations

This study had several limitations of note. First, a stated objective of PREFER was to make recommendations about how patients could be involved as research partners in preference research. As a result, PREFER project members (including all of the authors, of which MF, IH, RJ, SO, GS were scientific case study leads) were potentially biased in that they believed in the value of PPI and that it should be a standard component in preference research. Despite this potential for bias, however, results revealed not only positive impacts of PPI but negative effects on the research process as well, such as extra project time needed to train and support them.

Second, our sample size was small and restricted to the 8 PREFER preference case studies, thus potentially limiting the generalizability of our findings. However, aside from the core case studies, which were funded and conducted exclusively by the PREFER consortium, the other studies were conducted by either pharmaceutical companies or academic institutions, a mix which is reflective of the types of preference studies reported in the peer-reviewed literature to date.

Third, our analysis was a post-hoc evaluation of the PREFER case studies based on the final case study reports and supplemented by follow up questionnaires to the case study leads. As a result, our analysis was restricted to assessing qualitative data on researcher reports on impact. The primary purpose of these final study reports was to provide a comprehensive documentation of the scientific design, conduct and results of the cases studies; in contrast, documenting the role of patients as research partners was a secondary objective. However, qualitative data are vital for understanding a topic in depth as they provide detailed, contextual insights which quantitative data alone cannot provide. This approach allowed us to summarize and share researcher experiences of PPI across PREFER case studies with the intent of informing future preference studies.

Fourth, the PCORI framework, which we used as a conceptual guide to assessing PPI impact, had several limitations. While it was based on a review of the published and gray literature between 1995 and 2013, it has not been updated since initial development to include new findings in this field. Also, the state of the science in PPI research at that time featured primarily qualitative studies of patient engagement that focused on immediate and nearer term impacts only. Nonetheless, it remains a relevant and highly useful heuristic, and is consistent with findings from more recent reviews [15, 35]. Notable strengths of the PCORI framework are that it synthesized the relevant literature on hypothesized impacts of engagement and mapped them to what has actually been evaluated and assessed in the literature. In doing so, the authors identified seminal papers based on triangulating multiple data sources, and focused on studies which provided detailed, in-depth evaluations of collaborative approaches to patient and stakeholder engagement [19].

Fifth, we assessed only the reports of the preference study scientific case leads. Future research on this topic is needed to obtain the perspectives of patient research partners as well so that findings can be triangulated across these two different perspectives.

Not least, it is worth noting that the primary focus of these final study reports was on providing a detailed documentation of the scientific conduct and results of the case studies. In contrast, a describing the role of patients as research partners was a secondary objective.

## Conclusions

We found considerable heterogeneity across the case studies in terms of both the type and degree of PPI as well as the type and number of impacts, and barriers and facilitators observed. This variability suggests that patient research involvement was not accorded equal priority and attention across the 8 case study teams. Several factors may have contributed to this situation including the demanding pace of study timelines (which created pressure to prioritize study execution) and differences among the case study leads in degree of knowledge and expertise in working with patient partners. In particular, we found that that the number of positive impacts was higher in instances where the leads had had prior experience working with patient partners, had expressly stated the purpose for PPI in their study, and possessed pre-existing relationships with their patient partners.

A substantial number of unique barriers to PPI were identified. These barriers highlight gaps in the implementation of PPI within this group of preference studies. These results suggest that multi-faceted implementation strategies may be needed to support adoption, integration and sustainment of PPI efforts in the context of preference research. The selection of specific strategies to use can be informed both by the facilitators identified in this study and by the application of implementation science frameworks [42].

Additional detailed, case study-based examples of patient partner involvement in preference research would be valuable, studies that not only characterize the types of activities performed but the degree to the patient partner involvement in project decision-making and whether and what extent their input was accepted and used by the research team. One way to encourage more reporting of this type would be to require that PPI be evaluated as a standard component of preference research studies [19].

To advance the science of PPI, it would be useful to compare participants’ experiences in preference studies with and without PPI (e.g., in terms of understandability of the choice tasks and the different attributes, and the ease with which they were able to complete the preference questionnaire). Such comparison studies could also examine whether there were differences in data quality across studies without and without PPI. There is a need as well to establish consensus on key PPI terminology, including the definition of the patient partner role, and to use psychometrically validated measures of impact [43]. Not least, the quality, consistency and transparency of reporting on PPI should be enhanced via the use of quality reporting guidelines [44]. From a study operations standpoint, there is also a need for a training curriculum for patient preference study investigators on how to involve patients as research partners, and guidance on how to plan for supporting their sustained involvement.

Collectively, such efforts could advance both the science and practice of patient preference research and ensure that the promise of patient-centered decision-making regarding medicinal therapies is more fully realized.

## Supplementary Information

**Additional file 1.** Follow-up Questionnaire on barriers and enablers to patient involvement as research partners.

**Additional file 2: Fig. S1.** Framework for identifying where patients can be involved in patient preference research.

**Additional file 3.** Description of the PREFER Case Studies: Scientific aims, country, number of patient research partners, and activities performed.

## Data Availability

All data generated or analysed during this study are included in this published article.

## Abbreviations

COPD: Chronic Obstructive Pulmonary Disease
HTA: Health Technology Assessment
IMI-PREFER: Innovative Medicines Initiative (IMI- The Patient Preferences in Benefit-Risk Assessments during the Drug Life Cycle (PREFER)
MM: Multiple Myeloma
NMD: Neuromuscular Disorder
NSCLC: Non-Small Cell Lung Cancer
OA: Osteoarthritis
PAG: Patient Advocacy Group
PCORI: Patient-Centered Outcomes Research Institute
PPI: Patient and Public Involvement
RA: Rheumatoid Arthritis
