# Supplementary material for: Patients as research partners in preference studies: learnings from IMI-PREFER
Source: Res Involv Engagem. 2023 Apr 7;9:21. doi: 10.1186/s40900-023-00430-9 (PMC10080166; doi:10.1186/s40900-023-00430-9)
Supplement: Supplementary file 1 — Additional file 1. Follow-up Questionnaire on barriers and enablers to patient involvement as research partners. [file 40900_2023_430_MOESM1_ESM.pdf]

Additional file 1: Questionnaire on barriers and enablers to patient involvement as research partners in preference studies

Patient Involvement as Research Partners - PREFER Case Studies

We are developing a manuscript describing the involvement of **Patients as <u>Research Partners</u> (PRPs)** in the PREFER case studies. We are seeking your input regarding factors that served as **barriers and enablers** for PRP involvement along the process of your case study. This survey should not take you more than 5-7 minutes to complete.

1.Which case study would you be completing this survey for?

- ◯ COPD
- ◯ RA-Uppsala
- ◯ RA First Degree Relative
- ◯ PAVING (gene therapy)
- ◯ Diabetes
- ◯ MSD-Heart
- ◯ NMD
- ◯ Multiple Myeloma
- ◯ Lung Cancer
- ◯ OA Pain
- ◯ Hemophilia

2.Did your case study involved patients as partners or patient representatives as part of the project development?

- ◯ Yes
- ◯ No

3.How many patients or patient representatives were included in your research as partners?; AND please described what type of patient representatives they were (e.g., patients, caregivers, PAG representatives, etc.)

4.Can you please describe the type of activities and/or decisions in which your patient partners got involved with?

5.Which would you say was the added value of their involvement?; AND, was there any key decision throughout the project influenced by the input from your patient partners?

6.Please indicate in the list below **barriers to having Patients as Research Partners** involved in your case study:
Please mark all those that apply and feel free to add in additional as needed - 'other'

- [ ] B-PL =Team used technical/medical research terminology as opposed to a common 'plain language' approach
- [ ] B-RESIST = Researchers resisted/undermined Patient Research Partner (PRP) involvement
- [ ] B-CONTENT = PRP lacked sufficient content knowledge to contribute
- [ ] B-DYNAMICS= Research team group dynamics were negative/dysfunctional
- [ ] B-TENSION= There was tension between scientific research leads and PRPs
- [ ] B-POWER= PRPs perceived power/authority differential between them and the other research team members
- [ ] B-UNCERTAIN= There was uncertainty regarding how to practically operationalize the role of Patient as Research Partner (PRP)
- [ ] B-IMPRECISE= The role of the PRP was not clearly defined/no job description provided and no rules of engagement were presented
- [ ] B-TIME= Insufficient time to get Patients as Research Partners fully involved- focus was on getting the research project up and running
- [ ] B-GEO = Geographical limitations for Patients to participate as PRPs
- [ ] B-SUPPORT=Inadequate resources were allocated (e.g., funding to pay for PRPs; formal on-boarding; training; other sources of support)
- [ ] B-PANDEMIC=COVID-19 pandemic interfered with study execution and/or PRPs ability to contribute to study
- [ ] B-RECRUIT_PAG- Having to rely on the Patient Organization for Recruitment purposes - delays, non-responsive, couldn't get sufficient patients
- [ ]

7.Please indicate in the list below **enablers to having Patients as Research Partners** involved in your case study:
Please mark all those that apply and feel free to add in additional as needed - 'other'

- [ ] E-PURPOSE= Clearly stated purpose for involving Patients as Research Partners

☐ E-RELATION= Presence of existing (informal) relationships were in place between research team members and the Patients who served a Patient Research Partners (PRP)

☐ E-EXPERIENCE = PRP had participated in prior research studies

☐ E-REPRESENT= There was more than 1 Patient as Research Partner (PRP) involved in the study

☐ E-OWNER= There was a clear sense of 'co-ownership' between PRPs and research team members in terms of the research agenda

☐ E-URGENT= There was a sense of urgency about the importance of doing the research

☐ E-TIME= There was sufficient lead time to involve/recruit patients as PRPs

☐ E-MEETING=Research team meetings were scheduled to accommodate PRPs

☐ E-SUPPORT=Principal investigator of study ensured that PRPs received information, training to support them in their role

☐ E-MECHANISM=The research project work was organized in such a way as to ensure that the patient's voice was incorporated

☐ E-RECRUIT= Patients as Research Partners (PRPs) were recruited via networking with existing patient stakeholder groups

☐ E-PAY= Patients as Research Partners received fair financial compensation for their work as PRPs

☐ E-CONTRIBUTE= Patient contributions as PRPs was encouraged/recognized by the research time

☐

8.If you could **go back and re-conduct** your PREFER case study, what would you do differently in regard to involving Patients as Research Partners?

9.Did you provide any type of payment (actual money or some other type of compensation or incentive) to your Patients as Research Partners for their involvement?

○ Yes, we provided actual money AND an other type of compensation

○ Yes, actual money only

○ No actual money but we provided an other type of compensation

○ No incentives

○
