# Supplementary material for: Patients as research partners in preference studies: learnings from IMI-PREFER
Source: Res Involv Engagem. 2023 Apr 7;9:21. doi: 10.1186/s40900-023-00430-9 (PMC10080166; doi:10.1186/s40900-023-00430-9)
Supplement: Supplementary file 2 — Additional file 2: Fig. S1. Framework for identifying where patients can be involved in patient preference research. [file 40900_2023_430_MOESM2_ESM.pdf]

Additional file 2.

Figure 1. Framework for identifying where patients can be involved in patient preference research from Van Overbeeke et al., 2020. Reproduced without changes under Creative Commons Attribution ("CC BY") licence (http://creativecommons.org/licenses/by/4.0/).

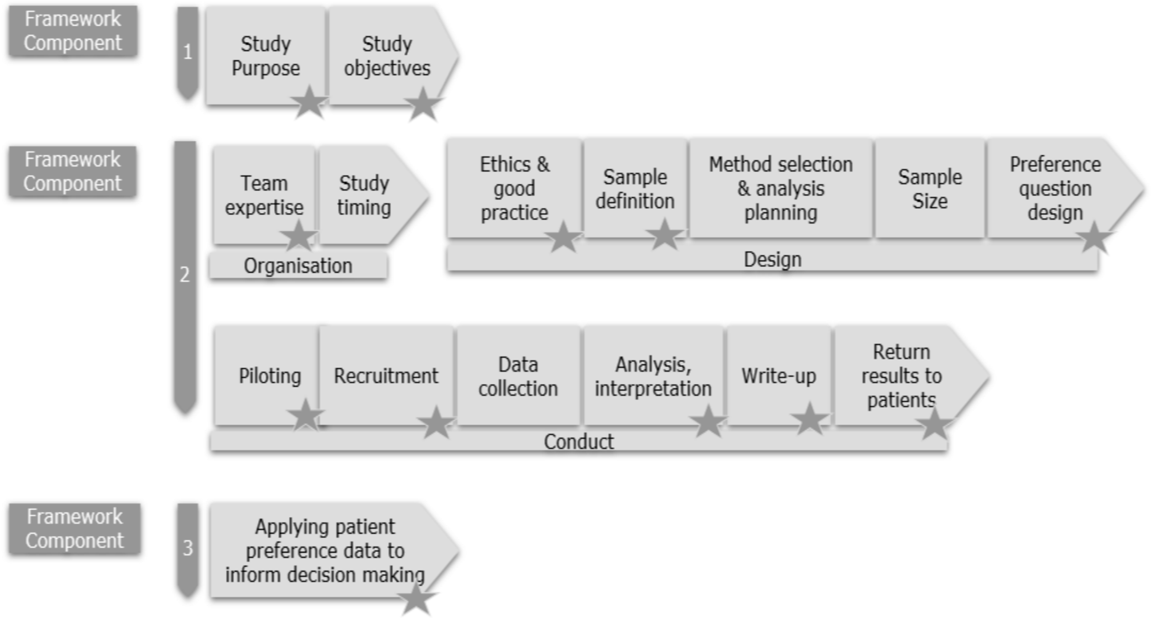

Legend: Star symbol indicates where patients can be involved as research partners in preference studies.
