# Supplementary material for: Patients as research partners in preference studies: learnings from IMI-PREFER
Source: Res Involv Engagem. 2023 Apr 7;9:21. doi: 10.1186/s40900-023-00430-9 (PMC10080166; doi:10.1186/s40900-023-00430-9)
Supplement: Supplementary file 3 — Additional file 3. Description of the PREFER Case Studies: Scientific aims, country, number of patient research partners, and activities performed. [file 40900_2023_430_MOESM3_ESM.pdf]

Additional File #3: Description of the PREFER Case Studies: Scientific aims, country, number of patient research partners, and activities performed.

	COPD	Gene Therapy for Hemophilia	Hemophilia	MM	NMD*	NSCLC*	RA	RA Preventive Treatment*
Scientific Aims	To quantify the relative importance that COPD patients place upon different disease-related endpoints (both novel and traditional).	To understand the willingness of adult patients with hemophilia A or B to make trade-offs between standard [illegible] care and gene therapy.	To identify the attributes of hemophila treatment that patients viewed as most important; and determine the willingness of hemophiliacs to trade-off specific risks in exchange for specific benefits.	To determine the treatment attributes of importance to MM patients; and to quantify the relative value of these different attributes to MM patients.	To determine unmet treatment needs of neuromuscular disease patients with either myotonic dystrophy type 1 (DM1) and mitochochrondrial myopathies (MM): and, to determine the treatment attributes of importance to these patients and their willingness to trade-off specific risks in exchange for specific benefit(s) [illegible] treatment.	To determine treatment attributes and the level of risk non-small cell lung cancer (NSCLC) patients might be willing to tolerate in exchange for a certain benefit in the context of available immune-therapy versus chemo-immunotherapy options.	To identify the most important treatment attributes for RA patients; and, to quantify patients' willingness to trade off specific risks in exchange for specific benefit(s).	To establish the preferences of 'at risk' individuals and the general public about preventive treatments for RA and their willingness to trade-off risks for benefits.
Country/ies where study was conducted	Australia, France, Japan, United Kingdom (UK), United States (US)	Belgium	UK	Belgium, Finland, Romania, Spain	UK	Belgium, Italy	Sweden	Germany, the Netherlands, Sweden, UK
Number of Patient Research Partners (PRPs)	2	2	4	9	4	3	2	8
Study Lead had experience with involving patients as research partners	No	No	No	No	Yes	No	Yes	Yes
Activities/tasks PRPs performed	PRPs provided input on wording of patient survey instrument; recruitment of study participants; input regarding the different disease attributes (e.g., mucus) and which are most challenging which helped in wording of final	PRPs provided input on wording of patient survey and other patient-facing materials to improve understandability; piloted the survey; and co-wrote lay summary of study findings.	One of the PRPs helped with recruitment by identifying patient advocates and caregivers who were then involved in the qualitative part of the study. The PRPs reached out to their Patient Advocacy Group	**Qualitative Phase**: PRPs provided advice on duration of the focus group and in the wording of patient-facing materials to reduce cognitive	PRPs participated in team meetings as a disease consultant; input on wording of patient-facing study materials (e.g, educational materials); participated in pre-testing of	PRPs advised on patient recruitment to both qualitative and quantitative studies; reviewed and commented on qualitative and quantitative study results; assisted in reviewing and disseminating Plain Language	PRPs were involved in identifying, selecting and framing of the treatment attributes and attribute levels. They provided input on the content and wording of the patient educational materials, and assisted in the	PRPs assisted in developing the funding application for the study, commented on draft versions of the application, and participated in preliminary consortium meetings. They advised on PREFER

	attribute description and related questions in the overall survey.		membership to recruit participants for the quantitative phase (DCE).<br>They also advised on content of recruitment emails and distributed these to their membership, delivering a series of recruitment communications to encourage participation.<br><br>**Qualitative study:** Interviews were held with 12 UK patients advocate PRPs and 2 UK caregiver RPPs to identify important attributes and levels to be used for eliciting patient preferences.<br>**Quantitative:** 2 UK PRPs reviewed the language/wording of the final chosen attributes and level descriptions (arising from the qualitative phase) from a health literacy perspective to ensure wording was clear and appropriate for people with haemophilia in the UK. They also reviewed the draft informed consent, patient online survey/questionnaire, and the text and images for an	burden and improve understandability; input in analysis of qualitative data and return of results back to patient community, including co-writing of the accompanying manuscript, abstracts and posters.<br>**Quantitative phase:** Input on wording of attributes, levels and preference elicitation questions; reduced length and cognitive burden of survey; improved clarity of Informed Consent; assisted with recruitment; provided input on analysis and interpretation of study results; helped disseminate study methodology and results to patient community; contributed to development of final research paper, abstracts and posters;	survey; input into survey design; assisted in development of a lay ("Plain Language") summary of study results and disseminated it back to the patient community. One PRP participated in a panel at one of the PREFER annual meetings.	Summary of quantitative study results.	design of the digital educational tool. They provided input into the content and wording of the quantitative survey and participated in the pre-testing of the survey instrument. They helped interpret study results and provided input into the discussion of results in the final report.	funding application development. They helped develop the clinical objectives of the quantitative study.<br>They contributed to content and design of focus group schedule<br>Contributed to analysis and interpretation of qualitative data<br>Involved in attribute selection<br>Contributed to survey development (content and format) and survey pre-testing<br>Attended(virtual) meetings<br>Provided feedback on slides for various meetings<br>Co-authors qualitative paper and lay summary<br>Involved in writing the PREFER recommendations<br>Co-presenter at RA case study webinar.

			educational video on gene therapy to be used in the quantitative study. The PRPs reviewed these materials on their own then consulted with external health literacy experts to discuss and agree on revisions. Final suggestions for revisions were then incorporated into these documents.	disseminated study findings back to patient community to support disease advocacy efforts.				

* Denotes PREFER core case study.
